# Identifying Gaps in Predoctoral Craniofacial Education

**DOI:** 10.3390/dj13060266

**Published:** 2025-06-16

**Authors:** Catherine Bingham, Linda Sangalli, Kathryn Preston, Poojan Shrestha, Caroline M. Sawicki

**Affiliations:** 1 Adams School of Dentistry, Chapel Hill, NC 27517, USA; cgb@ad.unc.edu (C.B.); poojansh@live.unc.edu (P.S.); 2 College of Dental Medicine–Illinois, Midwestern University, Downers Grove, IL 60515, USA; lsanga@midwestern.edu; 3 Solo Practice, Phoenix, AZ 85034, USA; kapres2@g.ucla.edu; 4 Department of Pediatric Dentistry and Dental Public Health, Adams School of Dentistry, Chapel Hill, NC 27517, USA

**Keywords:** predoctoral education, craniofacial abnormalities, dental education

## Abstract

**Background/Objectives:** It is essential that dental school graduates are adequately prepared to provide care to patients with craniofacial differences (PCD). This study aimed to identify potential educational deficiencies in predoctoral dental school curricula regarding the management of PCD. **Methods:** An electronic survey was distributed to predoctoral dental students across the United States. The 20-item questionnaire assessed students’ educational experiences, clinical encounters, and perceived knowledge and confidence in managing PCD. **Results:** The most taught didactic topic was diagnostic characteristics of craniofacial differences (77.1%), followed by psychosocial challenges (43.0%) and treatment/referral (36.3%). Respondents reported low levels of understanding and confidence in managing craniofacial conditions, with the lowest confidence in providing surgical treatment (30.1 ± 27.9) and the highest in referrals and communication (41.7 ± 30.1, on a 0–100 scale). Logistic regression showed that overall understanding was a significant predictor of confidence, increasing odds by 8% (OR = 1.08, 95% CI 1.05, 1.12). Participants noted that hands-on clinical experience would most improve their confidence in managing PCD. **Conclusions:** Predoctoral dental students exhibit low confidence and understanding in managing PCD. Incorporating more targeted craniofacial education, particularly hands-on clinical experience, into the curriculum is essential to better prepare dental graduates for craniofacial care.

## 1. Introduction

A multidisciplinary, team-based approach is the standard of care for treating patients with craniofacial differences (PCD), including those with congenital or acquired anomalies affecting the structure, function, or appearance of the head, face, and/or jaw [1]. However, only 205 craniofacial clinics approved by the American Cleft Palate Craniofacial Association (ACPA) exist across the U.S. [2]. While many patients live near these specialized centers, regular access to team-based care remains a challenge for the many who do not [3]. A study in North Carolina revealed that the average one-way travel time to primary craniofacial care was over an hour and a half, ranging from 5 to 480 min [4]. In order to address geographic barriers and reduce the burden on craniofacial teams, dental providers should ideally be able to collaborate with the patient’s craniofacial center to provide basic routine, supportive, and emergency care when indicated.

Dental providers are the healthcare professionals who identify and treat oral manifestations and dental development issues related to craniofacial conditions such as cleft lip and palate, craniosynostosis, hemifacial microsomia, Pierre Robin Sequence, and more [5]. Unfortunately, a large-scale survey of parents with children with special healthcare needs revealed that children with craniofacial differences had more unmet dental needs than children with other types of special health care needs [6]. This disparity can be explained by the greater number of barriers encountered such as difficulty finding a dentist willing to treat their child with a craniofacial difference, challenges locating a nearby provider, limited availability of convenient appointment times, and more [6]. The dental profession has the ability to improve access to care for this population by empowering dental providers to collaborate with the patient’s craniofacial team to deliver coordinated and appropriate care as needed.

To address these needs, it is essential that dental graduates understand their role in treating and referring PCD, as well as the associated anatomic, functional, aesthetic, and emotional challenges. To provide the standard of care, dental graduates should be prepared to collaborate with specialists, provide pre- and post-operative care, prevent oral disease, and understand craniofacial surgeries and their timing so that dental care can be carefully integrated into the overall treatment plan. Unfortunately, a lack of confidence in treating PCD has been reported overseas amongst general dentists, and it is likely that similar feelings of unpreparedness may be prevalent within the U.S. as well [7].

Currently, structured education in craniofacial care is primarily found in postgraduate specialty programs such as prosthodontics [8], pediatric dentistry [9], oral and maxillofacial surgery [10], and orthodontics [11], all of which have defined Commission on Dental Accreditation (CODA) standards that require clinical experience and didactic instruction in this area. These programs offer advanced training to manage the complex needs commonly seen in patients with craniofacial differences, yet these same patients also present in general practice settings, where care may be delivered by general dentists lacking similar preparation.

According to the ACPA Parameters of Care, general dentists are expected to treat the complex dental needs of this population and understand their associated medical and developmental comorbidities [5]. However, this expectation may be unrealistic without clear educational mandates at the predoctoral level. Despite the CODA requirements stipulating training in “special care dentistry”, there are no specific mandates for education related to craniofacial care in undergraduate dental programs [12]. PCD may present with challenges such as altered anatomy, surgical sites, craniofacial prosthetics, or psychosocial needs that require careful consideration. Without targeted education, graduates may feel unprepared to provide optimal care, potentially leading to disparities in access and quality of dental services for these patients.

The aim of this study was to assess U.S. dental students’ confidence and knowledge levels related to craniofacial care and identify potential educational gaps in predoctoral curricula. It was hypothesized that dental students would lack confidence in their role as general dentists providing care to the craniofacial population. Results of this study will highlight areas of weakness in the current dental curricula that, if addressed, have the potential to equip dentists with the necessary skills to adequately provide quality care to PCD. By fostering these collaborative efforts, general dentists can play a vital role in reducing disparities in care and improving overall treatment outcomes for PCD.

## 2. Materials and Methods

### 2.1. Study Design

This cross-sectional study was reviewed by the University of North Carolina Institutional Review Board (IRB) and deemed exempt (IRB#24-1907, 8 August 2024). An anonymous online questionnaire was distributed via Qualtrics to all predoctoral students from 70 CODA-accredited dental programs in the United States (approximately 23,000 students) from August 2024 to November 2024. The survey was disseminated via multiple channels, including the American Student Dental Association chapter presidents, American Dental Education Association chapter presidents, academic deans, and peer networks, ensuring broad reach to dental students. Any dental student, first through fourth year, enrolled in a predoctoral program during the study period was eligible and invited to participate. There were no restrictions on age, sex, or racial/ethnic composition. No formal randomization process was employed, as participation was voluntary and open to all eligible students, reflecting a non-probability sampling approach typical in survey research aiming for wide accessibility rather than randomized selection. Electronic informed consent was obtained from respondents prior to participation. In accordance with local IRB regulations, participants were not obligated to respond to all survey items.

### 2.2. Study Size

A prior sample size calculation was performed to determine the necessary sample size based on understanding and confidence levels related to PCD. Following a recommendation from behavioral survey studies of a sample-to-item ratio of 15:1 [13,14], a survey comprising 15 items would require a minimum of 220 participants. To enable meaningful outcome comparisons across different groups, each subgroup should include at least 30 participants, as supported by the Central Limit Theorem [15].

### 2.3. Survey Assessment Tool

The anonymous survey (Supplementary File S1) was co-developed by a craniofacial orthodontist (K.P.) and two pediatric dentists (P.S. and C.M.S.) who are specialized in craniofacial orthodontics and pediatric dentistry, respectively. Survey content was also reviewed by external content experts to ensure the relevance and appropriateness of questions, as well as readability and accessibility. The final survey consisted of 20 items across two sections. Responses to all sections were optional. Section 1 assessed the presence of didactic teaching (on diagnosis, psychosocial challenges, treatment, and referral) and clinical encounters related to craniofacial education. Section 2 evaluated perceived knowledge (7 items) on specific craniofacial conditions (orofacial clefts, Treacher Collins syndrome, craniosynostosis, cleidocranial dysplasia, Pierre Robin sequence, hemifacial microsomia, and Apert syndrome) and confidence levels (8 items) related to treating PCD by asking the participants to rate their knowledge and confidence levels on a 0–100-point scale (with 100 reflecting the highest scores). Investigated domains included confidence in providing regular care, restorative, surgical treatments; distinguishing between the need to treat or refer; communicating with other healthcare providers; interpreting radiographs; performing occlusal analyses; and educating patients and their families about oral health implications. Educational resources to improve confidence levels were also inquired about. Opportunities to elaborate on closed-ended responses were provided throughout via optional open text boxes, and the last item of the survey was an open text box with an invitation to share any other thoughts or feedback related to craniofacial education. The survey also assessed sociodemographic (e.g., age, sex, race, ethnicity, and academic year) characteristics.

### 2.4. Statistical Analysis

Data were inspected for missing values prior to analysis. Baseline demographics and all relevant variables were compared between participants with and without missing data to assess potential predictors of missingness. Missing data were assumed to be Missing at Random (MAR), and casewise deletion was employed for the analyses. Normality of outcome variables was assessed with the Shapiro–Wilk test, and logarithmic transformations were applied as needed to meet the assumptions of parametric tests.

Confidence and understanding levels were compared across academic years with analysis of variance (ANOVA, with Bonferroni post hoc tests) and between students with and without previous didactic training using independent *t*-tests. To minimize the risk of type I error from multiple comparisons, the Holm–Bonferroni correction was applied. An overall confidence index and understanding index were calculated as the average of scores across the eight confidence-related items and the seven understanding-related items, respectively. Logistic regression analyses were performed to examine whether academic year, prior didactic training, and self-perceived understanding level were significant predictors of sufficient overall confidence (defined as >50% on a 0–100 scale). A similar logistic regression was conducted to investigate whether academic year, prior didactic training, and self-perceived confidence level predicted sufficient overall understanding (defined as >50% on a 0–100 scale). For those variables identified as significant predictors in the logistic regression analysis, a moderator analysis was conducted to examine the level of strength between independent and dependent variables. Finally, cluster analysis was conducted to identify student groups based on their confidence and understanding levels.

Data analyses were conducted using SPSS (IBM (Armonk, NY, USA), SPSS Inc. (Chicago, IL, USA), v.27) and SAS^®^ OnDemand for Academics (version 9.4, SAS Institute Inc., Cary, NC, USA). Significance level was set at 0.05.

## 3. Results

From August to November 2024, a total of 275 predoctoral dental students participated in this study. After excluding incomplete responses (N = 48), the final analysis included 227 predoctoral dental students (25.6 ± 3.4 years old, 69.6% females). Table 1 summarizes the demographic characteristics of the sample. Participants were distributed across academic years as follows: first year (25.6%), second year (28.2%), third year (25.1%), and fourth year (21.1%).

**Figure 1:**
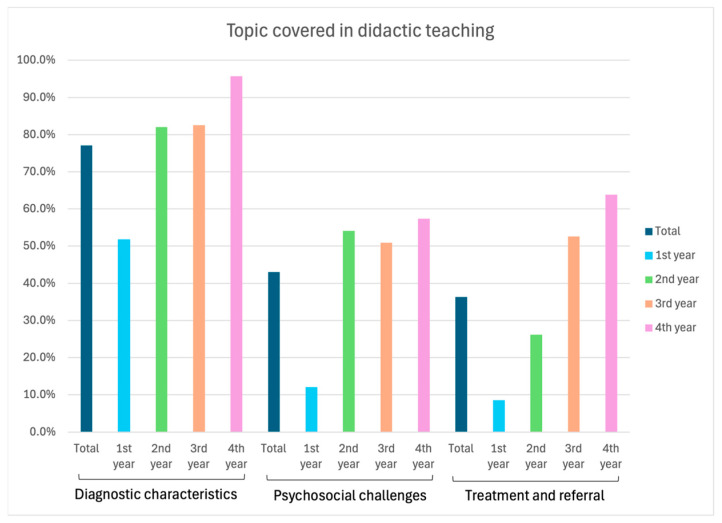
Distribution of didactic teaching across academic years.

The most taught topic in predoctoral curricula was the diagnostic characteristics of craniofacial differences (77.1%), followed by psychosocial challenges faced by PCD (43.0%) and the treatment and referral of these patients (36.3%). Statistically significant differences were observed in the distribution of these topics across academic years, with fourth-year students receiving most of the didactic instruction (*p* < 0.001, Figure 1).

### 3.1. Self-Perceived Confidence Levels and Impact of Prior Didactic Teaching

**Figure 2:**
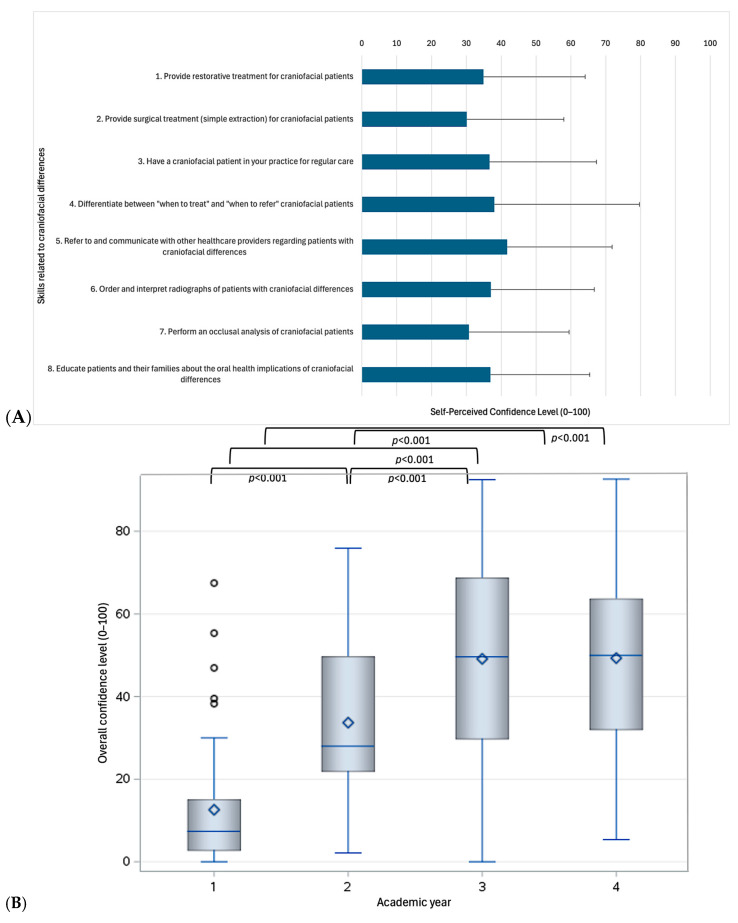
Self-perceived confidence level across all participants (**A**) and differences across academic years (**B**).

Using a 50% cut-off to define adequate confidence [16], students exhibited overall low to moderate confidence levels (ranging from 30.1 to 41.7 on a 0–100 scale, Figure 2A). Participants felt least confident in performing simple extractions on PCD (30.1 ± 27.9) and most confident in referring patients and communicating with other healthcare providers (41.7 ± 30.1 on a 0–100 scale). As expected, confidence levels showed broad variability and differed significantly by academic year (Figure 2B). Fourth-year students demonstrated significantly higher confidence levels compared to first- and second-year students across all domains assessed (*p* < 0.05), except for “referring patients and communicating with other healthcare providers” (*p* = 0.113) and “educating patients and their families about the oral health implications of craniofacial differences” (*p* = 0.728), where their scores were similar to those of second-year students. No significant differences were observed between third- and fourth-year students (*p* > 0.05).

**Figure 3:**
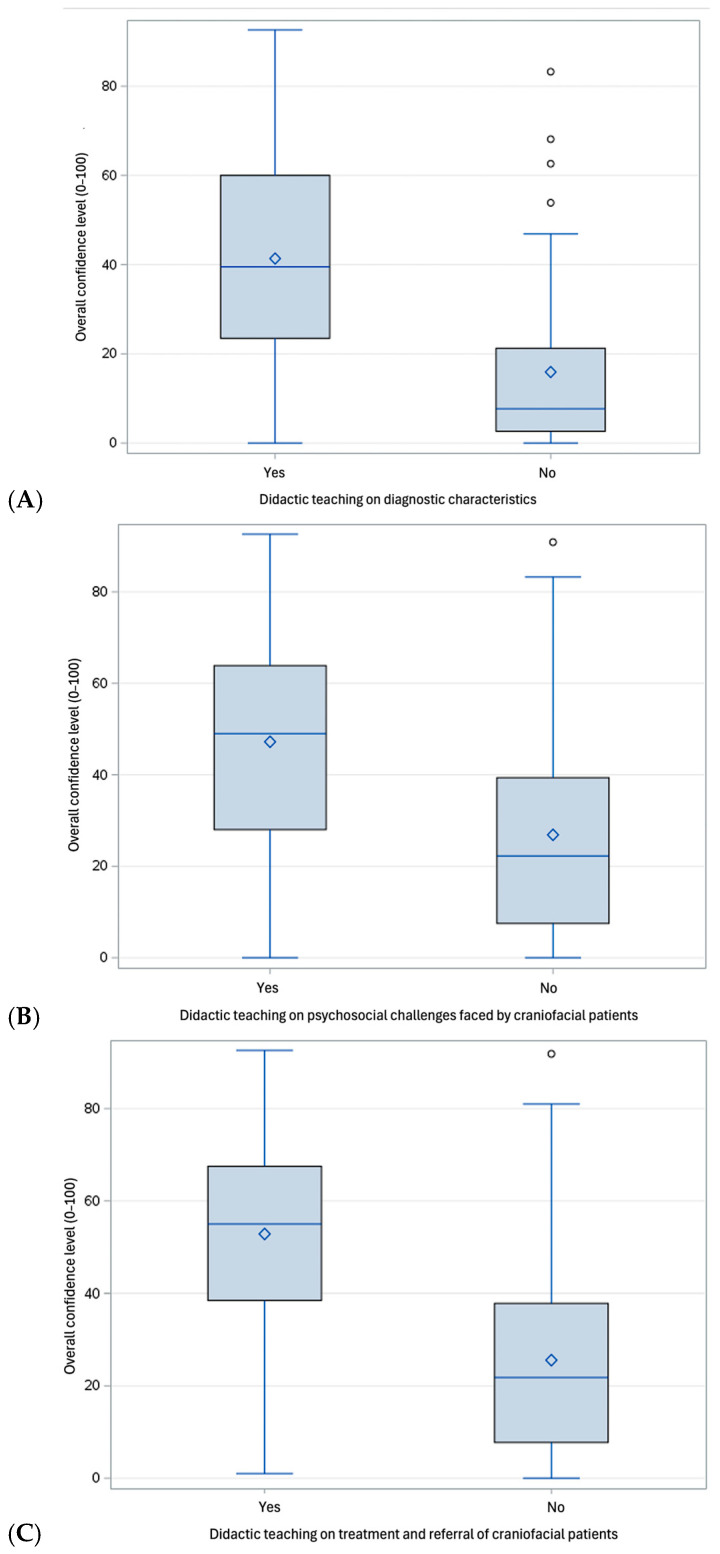
Self-perceived confidence level across all participants according to previous didactic teaching on diagnostic characteristics (**A**); psychosocial challenges faced by craniofacial patients (**B**); and treatment and referral of craniofacial patients (**C**).

Prior training (particularly in the treatment and referral, and, to a lesser extent, in psychosocial challenges faced by PCD) was significantly associated with overall higher confidence levels across all participants (*p* < 0.001, Figure 3A–C). Interestingly, previous didactic training was associated with higher confidence levels across several PCD-related skills among third-year students (Table S1) Similarly, previous didactic teaching was associated with higher confidence levels in ordering and interpreting radiographs of PCD and in patients’ education about oral health implications of craniofacial differences among second-year students (Table S1). Conversely, there was no significant difference in confidence level between students with and without previous didactic teaching among fourth-year dental students, where the confidence levels were perceived as relatively moderate across all participants (*p* > 0.05).

A strong positive correlation was observed between understanding craniofacial conditions and perceived confidence levels (*p* < 0.001, Table S2). Logistic regression identified overall understanding as a significant predictor of sufficient confidence levels (*p* < 0.001, Table 2), increasing the odds by 8% (OR = 1.08, 95% CI 1.05, 1.12). The model was statistically significant (X^2^ (5) = 28.262, *p* < 0.001), accounted for 60.8% of the variation in sufficient confidence level (Nagelkerke R^2^), and correctly predicted 86.3% of the cases. Academic year and prior didactic training were not significant predictors, except for prior teaching on treatment and referral, which increased the odds of sufficient confidence by 5.84 times (95% CI 2.28, 14.99). These effects were sustained in the analysis stratified by academic year (Table S3).

The moderator analysis revealed that receiving prior training in the treatment and referral of PCD did moderate the relationship between understanding and confidence level. Specifically, prior training increased the variation in confidence level by 5.6%, and this increase was statistically significant (*p* < 0.001).

### 3.2. Self-Perceived Level of Understanding and Impact of Prior Didactic Teaching

**Figure 4:**
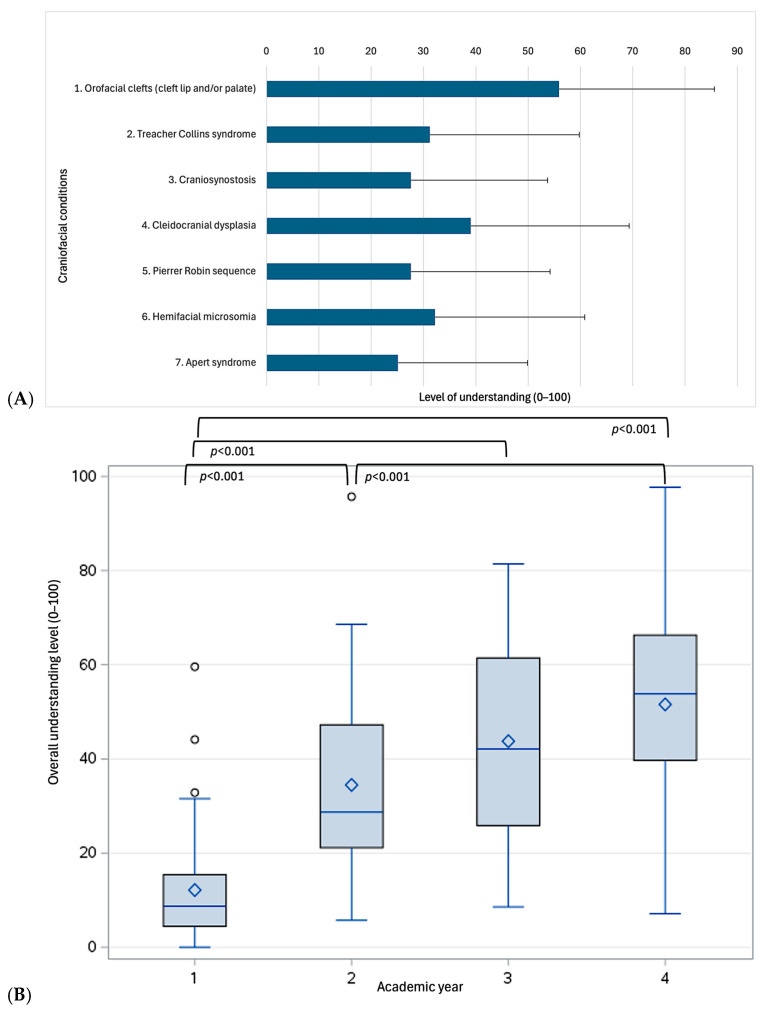
Self-perceived level of understanding across all participants (**A**) and differences across academic years (**B**).

Participants reported a relatively low level of understanding of craniofacial education (35.0 ± 23.6, ranging from 0.0 in two participants to 97.7 in one participant, with 7.1% scoring below 50%, Figure 4A). Significant differences were observed across academic years (Figure 4B). Specifically, fourth-year dental students scored significantly higher compared to first-year (51.6 ± 21.0 vs. 12.2 ± 12.1, *p* < 0.001) and second-year students (51.6 ± 21.0 vs. 34.5 ± 19.5, *p* < 0.001), while no significant difference was observed with third-year students (51.6 ± 21.0 vs. 43.8 ± 20.4, *p* = 0.227). Similarly, third- and second-year students did not significantly differ (*p* = 0.066).

**Figure 5:**
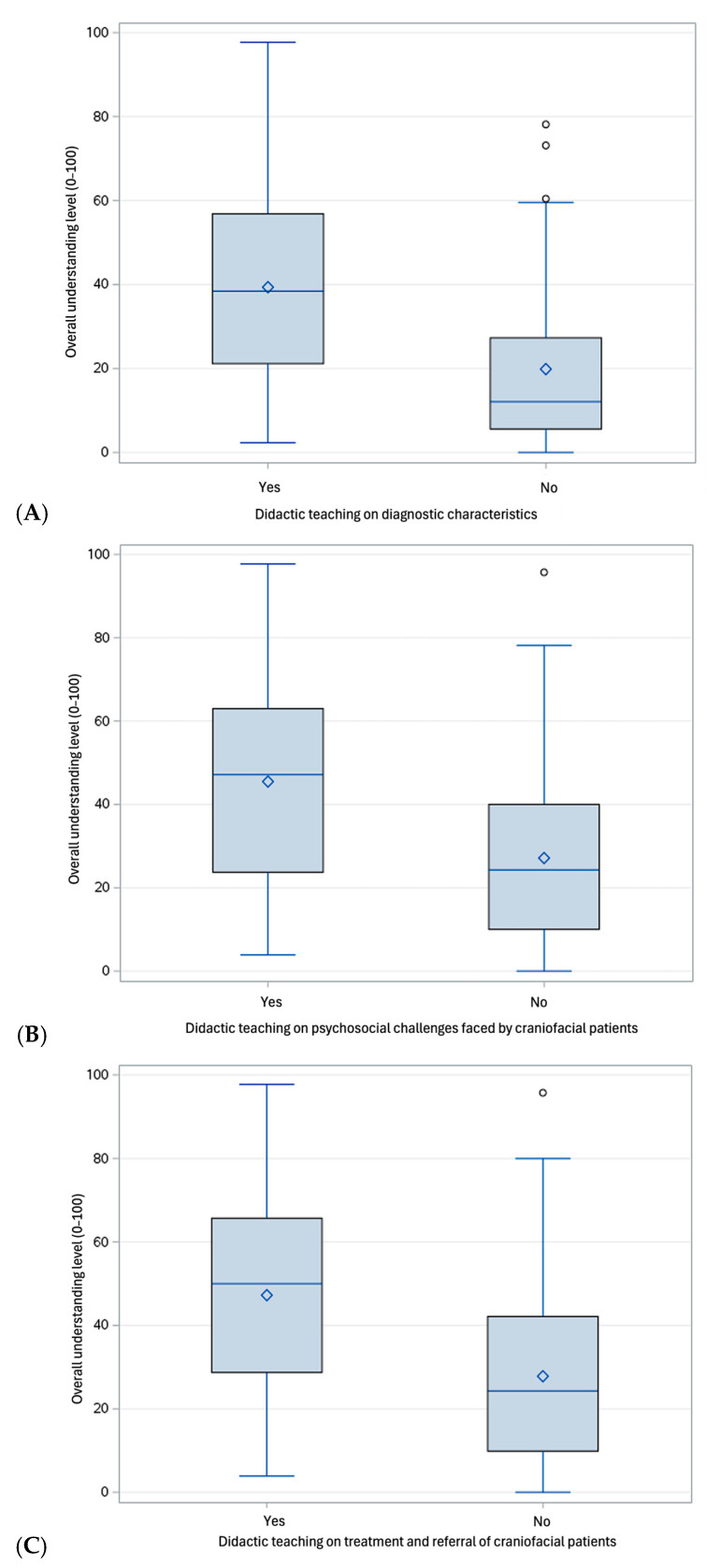
Self-perceived level of understanding across all participants according to previous didactic teaching on diagnostic characteristics (**A**); psychosocial challenges faced by craniofacial patients (**B**); and treatment and referral of craniofacial patients (**C**).

Prior training in diagnostic characteristics, psychosocial challenges, and treatment/referral of PCD significantly increased understanding across all participants (*p* < 0.001, Figure 5A–C).

Logistic regression identified that both overall confidence level (*p* < 0.001) and attending the fourth year (*p* = 0.034) were significant predictors of sufficient understanding, with OR = 1.05 (95% CI 1.03, 1.08) and OR = 10.74 (95% CI 1.20, 96.01), respectively (Table 2). The model was statistically significant (X^2^ (5) = 38.865, *p* < 0.001), accounted for 45.8% of the variation in sufficient confidence level (Nagelkerke R^2^), and correctly predicted 82.4% of the cases. Conversely, prior didactic training was not a significant predictor.

### 3.3. Cluster Analysis

**Figure 6:**
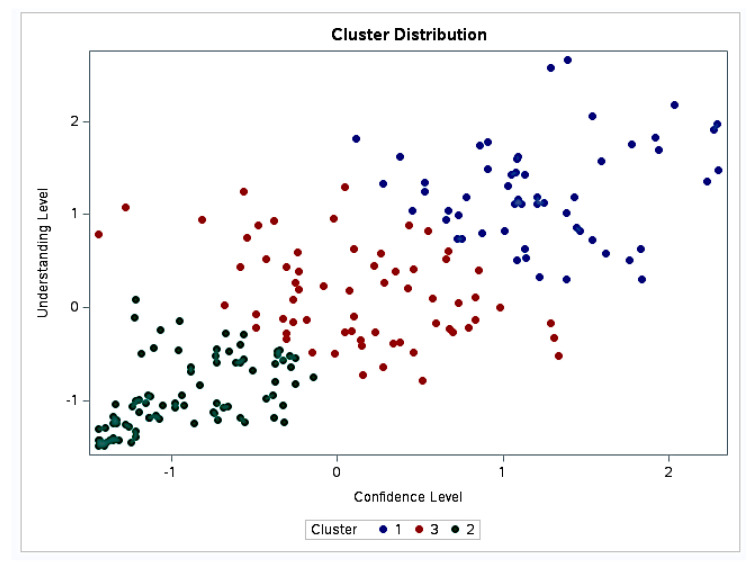
Cluster analysis based on the level of confidence and understanding.

The cluster analysis identified three groups (Figure 6). Group 1 (N = 56 participants) corresponded to the highest confidence (mean confidence level, standardized score = 1.24 ± 0.53) and understanding levels (mean understanding level, standardized score = 1.24 ± 0.53). Group 2 (N = 85 participants) was characterized by the lowest confidence (mean confidence level, standardized score = −0.91 ± 0.38) and understanding levels (mean understanding level, standardized score = −0.93 ± 0.38). Group 3 (N = 66 participants) included participants with moderate confidence (mean confidence level, standardized score = 0.12 ± 0.57) and understanding levels (mean understanding level, standardized score = 0.15 ± 0.51), roughly around the average for the dataset.

Educational resources perceived as most helpful in improving dental students’ confidence to provide care for PCD included hands-on clinical experience (85.1 ± 20.9), clinical rotations in specialized centers (82.4 ± 22.3), and shadowing opportunities (77.5 ± 25.2). Online modules (37.40 ± 29.57) and additional textbooks/articles (31.0 ± 27.15) were perceived as the least helpful resources for improving confidence to treat PCD.

## 4. Discussion

It is essential that dental graduates are adequately prepared to provide basic care to PCD, whether they plan to serve on specialized craniofacial teams or not. The current study revealed that dental students have a relatively low level of understanding of craniofacial conditions and low-to-moderate confidence in the basic management and treatment of PCD. Similar educational shortcomings have been documented globally. A study conducted in Saudi Arabia assessed newly graduated dentists’ knowledge of cleft lip and palate and identified significant gaps in understanding risk factors and post-surgical complications, emphasizing the need for improved education within dental curricula. This study concluded that because general dentists play such a critical role in cleft lip and palate management, enhanced training is necessary to optimize patient outcomes [17].

While 77.1% of respondents indicated they were taught about the characteristics of craniofacial conditions, only 36.3% were taught how to treat and refer PCD. This discrepancy raises an important question: How much value is there in teaching the characteristics of craniofacial conditions if students are not being adequately trained to provide comprehensive care for this population? This gap in education has proven to be an issue for other patient populations, such as those with temporomandibular disorders and special healthcare needs, where inadequate training has led to significant barriers and inefficient access to care [18,19]. For instance, insufficient training in TMD management during dental school resulted in dental graduates providing fragmented care and ineffective treatment, leading to underdiagnosis, mismanagement, and patient frustration [19]. As demonstrated in other patient populations, this gap in craniofacial education could have serious implications for both the quality of care that PCD receive and the students’ readiness to manage such cases upon graduation. Without sufficient training on craniofacial care, dental graduates are left ill equipped to address the needs of PCD, potentially leading to delays in care, inappropriate referrals, and decreased access to care.

Among the various aspects of care for PCD, dental students reported feeling most confident in referring these patients to other healthcare providers. While understanding how and when to make referrals is undoubtedly an essential skill, the fact that this is the area where students expressed the greatest confidence—rather than more direct care practices like restorative treatment or simple extractions—may suggest a lack of confidence in their ability to manage basic dental care for PCD themselves. Accordingly, previous studies in healthcare practitioners have demonstrated that higher referral rates are associated with lower levels of clinical confidence [20]. Referring to other specialists is an important aspect of providing comprehensive, multidisciplinary care for complex patients; however, it is equally critical that dentists feel empowered to provide the basic care they are qualified to offer in collaboration with a craniofacial team, rather than defaulting to immediate referral. With certain craniofacial centers having over 5000 active patients and receiving 500 new patient referrals annually, this balance between referrals and direct care is key to ensuring craniofacial clinics are not over-burdened and PCD receive timely and appropriate treatment [21].

Additionally, only 43% of respondents were provided training on the psychosocial challenges faced by PCD. This lack of education is concerning, considering that 70% of PCD over age 6 are affected by psychosocial problems [22]. Families of PCD are also vulnerable to these challenges, further highlighting the need for comprehensive psychosocial support [23]. ACPA guidelines strongly emphasize the importance of understanding the psychosocial aspects of craniofacial conditions and screening for psychosocial stressors, as these patients and their families often experience greater emotional difficulties [24]. Psychosocial training helps providers better empathize with the challenges these patients face and provides insights into how to communicate effectively with patients and their families, addressing both their clinical and emotional needs [25]. Correctly managing psychosocial issues can lead to better medical and psychosocial results, including enhanced adherence to treatment plans and overall improvements in quality of life [26]. Therefore, integrating more psychosocial education into predoctoral dental curricula is essential for preparing future dentists to offer comprehensive, compassionate care to the craniofacial population and their families.

Identifying educational gaps in predoctoral training offers dental schools a valuable opportunity to critically evaluate and enhance their curricula to better address these deficiencies. Improving predoctoral training in the basic oral health management of patients with craniofacial differences can better equip more general dentists to provide effective care, thereby reducing disparities in access and improving clinical outcomes. Furthermore, as proven in medical school settings, greater, more in-depth exposure to craniofacial education throughout dental school may inspire students to pursue related specialty training such as orthodontics, pediatric dentistry, prosthodontics, or oral and maxillofacial surgery with the intent to provide more advanced care to individuals with craniofacial conditions [27]. By strengthening foundational education, dental programs can expand both the general and specialty workforce capable of addressing the complex needs and access disparities of this population.

This study also provides insights into the educational resources that dental students perceive as most helpful in improving their confidence to provide basic care for PCD. Greater hands-on experience and active participation increase students’ preparedness, confidence, competency, and communication skills [28,29]. For instance, after the New York University College of Dentistry introduced special needs clinical rotations, predoctoral dental students reported increased self-efficacy in treating patients with special healthcare needs, including greater understanding of their barriers to care, improved confidence, and intentions to treat this population in future practice [30]. By incorporating more experiential learning experiences into the predoctoral craniofacial curricula, students would be provided with direct exposure to craniofacial care, allowing them to build the practical skills, clinical judgment, and confidence necessary for effective treatment. By prioritizing clinical rotations, shadowing, and other interactive learning formats, dental schools can better prepare their students to meet the needs of PCD with collaboration and direct care.

While this study confirms that improvements to predoctoral craniofacial curricula are indicated, developing a standardized, evidence-based framework will likely be challenging. This is largely due to the significant variability in diagnostic and treatment protocols within the cleft and craniofacial provider community itself [31]. Even among experienced providers, there is no universal consensus on the best practices for diagnosing and treating craniofacial conditions, complicating efforts to establish consistent care guidelines. Therefore, further research is needed to develop evidence-based protocols that can guide the creation of standardized craniofacial curricula and clinical care.

There exist several limitations when interpreting the results of this study. To begin, this study relies on self-reported data, which may not accurately reflect students’ actual knowledge or clinical abilities. Perceived confidence can differ from true competence, introducing subjectivity into the findings. Secondly, the survey did not collect information about which dental school each respondent attended, limiting our ability to determine how broadly representative our findings are across U.S. dental programs and preventing the evaluation of variation between schools and regions. Additionally, while there are over 23,000 dental students in the U.S., it is important to note that the results of this study reflect the experiences of only 227 students [32]. As such, these findings may not be generalizable to all U.S. dental students and programs. To continue, nonresponse bias is also possible, as those with more interest or experience in craniofacial care may have been more likely to complete the survey, potentially skewing the results. Furthermore, survey fatigue may have affected response quality, particularly toward the end of the questionnaire. Finally, this study only focused on predoctoral programs in the U.S., which may not be applicable to dental curricula outside the U.S. Despite these limitations, this study is the first to examine and compare students’ educational experiences, clinical encounters, and perceived knowledge and confidence in managing PCD.

Future iterations of this study could include surveying residents and practicing dentists to offer a more comprehensive understanding of how dental education translates into confidence and ability to provide care for PCD. Additionally, involving academic deans and craniofacial educators in future studies would provide deeper insights into the structure, detail, and effectiveness of existing predoctoral curricula. Finally, future studies could incorporate more objective assessments such as scenario-based exams, Objective Structured Clinical Examinations, or supervisor ratings of students’ ability to apply knowledge in clinical settings.

## 5. Conclusions

This study highlights critical gaps in predoctoral dental education regarding the care of PCD, especially in clinical training, psychosocial education, and practical skills development. Although students reported moderate familiarity with craniofacial conditions, their limited confidence in providing direct care highlights the need for improved educational approaches to better prepare dental graduates to manage PCD effectively. Addressing educational gaps through experiential learning, such as clinical rotations, could enhance students’ competence and confidence while reducing over-reliance on referrals to specialists. Ultimately, standardizing predoctoral craniofacial curricula and expanding evidence-based training opportunities is critical for future dental graduates to be better equipped to deliver comprehensive care for PCD and their families.

## Figures and Tables

**Table 1:** Demographic characteristics of all participants.

	All Participants (N = 227)
Age (mean, SD)	25.6 ± 3.4
Sex (N, %)	
Females	158 (69.6)
Males	65 (28.6)
Prefer not to say	4 (1.8)
Race	
Asian	58 (25.6)
White	128 (56.4)
African American	18 (7.9)
Native Hawaiian	1 (0.4)
Two or more races	5 (2.2)
Other	7 (3.1)
Prefer not to say	10 (4.4)
Ethnicity	
Hispanic/Latinx	22 (9.7)
Non-Hispanic	190 (83.7)
Other	4 (1.8)
Prefer not to say	11 (4.8)
Academic year	
First year	58 (25.6)
Second year	64 (28.2)
Third year	57 (25.1)
Fourth year	48 (21.1)

**Table 2:** Logistic regression analysis to predict sufficient confidence and understanding levels (both above 50% threshold) across all participants.

Predictors		Beta	SE	Wald	*p* Value	OR	95% CI LL, UL
SUFFICIENT CONFIDENCE LEVEL							
Overall understanding	Self-perceived level of understanding	0.08	0.01	32.53	<0.001 *	1.08	1.05, 1.12
Academic year (Year 1 as reference)	Year 2	0.33	0.91	0.13	0.721	1.39	0.23, 9.32
	Year 3	0.82	0.91	0.80	0.372	2.26	0.38, 13.50
	Year 4	−1.12	0.95	0.02	0.902	0.89	0.14, 5.76
Previous didactic teaching	Diagnostic characteristics of craniofacial differences	0.46	0.80	0.34	0.561	1.59	0.33, 7.53
	Psychosocial challenges faced by craniofacial patients	−0.20	0.49	0.17	0.684	0.82	0.32, 2.12
	Treatment and referral of craniofacial patients	1.76	0.48	13.46	<0.001 *	5.84	2.28, 14.99
SUFFICIENT UNDERSTANDING LEVEL							
Overall confidence level	Self-perceived level of confidence	0.050	0.01	19.94	<0.001 *	1.05	1.03, 1.08
Academic year (Year 1 as reference)	Year 2	1.68	1.12	2.27	0.132	5.36	0.60, 47.78
	Year 3	1.80	1.12	2.58	0.109	6.03	0.67, 54.07
	Year 4	2.37	1.12	4.51	0.034 *	10.74	1.20, 96.01
Previous didactic teaching	Diagnostic characteristics of craniofacial differences	−0.46	0.67	0.47	0.493	0.63	0.17, 2.35
	Psychosocial challenges faced by craniofacial patients	0.40	0.44	0.85	0.357	1.50	0.64, 3.52
	Treatment and referral of craniofacial patients	0.30	0.45	0.43	0.512	1.34	0.56, 3.24

* *p* < 0.05. SE: standard error; LL: lower limit; UL: upper limit.

## Data Availability

The original contributions presented in the study are included in the article; further inquiries can be directed to the corresponding author.

## Supplementary Materials

The following supporting information can be downloaded at: https://www.mdpi.com/article/10.3390/dj13060266/s1, Supplementary File S1: Survey Questionnaire; Table S1: Association between perceived confidence level and previous didactic teaching according to academic year; Table S2: Correlation matrix between levels of confidence and understanding; Table S3: Logistic regression analysis to predict sufficient overall confidence level (above 50% threshold) according to academic year.

## Abbreviations

The following abbreviations are used in this manuscript:

ACPA	American Cleft Palate Craniofacial Association
CODA	Commission on Dental Accreditation
PCD	Patients with craniofacial differences

## References

[1] Singh V.P., Moss T.P. (2015). Psychological impact of visible differences in patients with congenital craniofacial anomalies. Prog. Orthod..

[2] Association TACPC Find A Care Team. https://acpacares.org/find-a-care-team/?tax%5Bacpa_team%5D%5B0%5D=9.

[3] Brown M.I., Kuyeb B.K., Galarza L.I., Benedict K.C., Hoppe I.C., Humphries L.S. (2025). Travel Burden to American Cleft Palate and Craniofacial Association-Approved Cleft and Craniofacial Teams: A Geospatial Analysis. Plast. Reconstr. Surg..

[4] Cassell C.H., Krohmer A., Mendez D.D., Lee K.A., Strauss R.P., Meyer R.E. (2013). Factors associated with distance and time traveled to cleft and craniofacial care. Birth Defects Res. A Clin. Mol. Teratol..

[5] Parameters of Care for Evaluation and Treatment of Individuals with Cleft Lip/Palate and/or Other Craniofacial Conditions. https://njcraniofacialcenter.com/wp-content/uploads/2025/05/2024-ACPA_ParametersOfCare_Final.pdf.

[6] Nelson L.P., Getzin A., Graham D., Zhou J., Wagle E.M., McQuiston J., McLaughlin S., Govind A., Sadof M., Huntington N.L. (2011). Unmet dental needs and barriers to care for children with significant special health care needs. Pediatr Dent..

[7] Gallagher N. (2020). A general dental practitioner’s role in treating patients with a cleft lip and/or palate. Br. Dent. J..

[8] Commission on Dental Accreditation Accreditation Standards for Advanced Dental Education Programs in Prosthodontics. https://coda.ada.org/-/media/project/ada-organization/ada/coda/files/prostho.pdf?rev=f35706a957954de0a3029fb29f6d0936&hash=AD2E63F25D177007EDC2FD30A4132FB5.

[9] Commission on Dental Accreditation Accreditation Standards for Advanced Dental Education Programs in Pediatric Dentistry. https://coda.ada.org/-/media/project/ada-organization/ada/coda/files/pediatric_dentistry_standards.pdf?rev=7a6894bb318149cc8777f72f008fda3c&hash=6828EA6716FA4B7B46BBC5EFCFF12515.

[10] Commission on Dental Accreditation Accreditation Standards for Advanced Dental Education Programs in Oral and Maxillofacial Surgery. https://coda.ada.org/-/media/project/ada-organization/ada/coda/files/oms.pdf?rev=0a48434f84f94d358d052cac22cf3832&hash=EFCA3E6C011604571F2CF6440C1503C1.

[11] Commission on Dental Accreditation Accreditation Standards for Advanced Dental Education Programs in Orthodontics and Dentofacial Orthopedics. https://coda.ada.org/-/media/project/ada-organization/ada/coda/files/ortho.pdf?rev=0c6354090d544a40b8d3c1e2dd77a2cf&hash=C54FE1B20236632A7EF58A09866A2E12.

[12] Commission on Dental Accreditation Accreditation Standards for Dental Education Programs. https://coda.ada.org/-/media/project/ada-organization/ada/coda/files/predoc_standards.pdf?rev=f8e014de5e534fa0bd995c09ed27e8e3&hash=F4F7AF8C59776FA039114955E7B07305.

[13] Osborne J.W., Costello A.B. (2004). Sample size and subject to item ratio in principal components analysis. Pract. Assess. Res. Eval..

[14] Memon M.A.T.H., Cheah J.H., Thurasamy R., Chuah F., Cham T.H. (2020). Sample size for survey research: Review and recommendations. J. Appl. Struct. Equ. Model..

[15] Costello A.B., Osborne J. (2005). Best practices in exploratory factor analysis: Four recommendations for getting the most from your analysis. Pract. Assess. Res. Eval..

[16] Sawicki C.M., Sangalli L. (2024). Pediatric Dentists’ Practice Patterns in the Screening, Diagnosis, and Management of Temporomandibular Disorders. Children.

[17] Agha B., Helal N.M.S., Al-Khafaji T.J., Farie G.A., Basri O., Fleming P.S. (2023). Knowledge assessment on cleft lip and palate among recently graduated dentists: A cross-sectional study. BMC Oral. Health.

[18] Lim M., Liberali S.A.C., Calache H., Parashos P., Borromeo G.L. (2021). Perceived barriers encountered by oral health professionals in the Australian public dental system providing dental treatment to individuals with special needs. Spec. Care Dent..

[19] Yost O., Liverman C.T., English R., Mackey S., Bond E.C., National Academies of Sciences, Engineering, and Medicine, Health and Medicine Division, Board on Health Care Services, Board on Health Sciences Policy, Committee on Temporomandibular Disorders (TMDs): From Research Discoveries to Clinical Treatment (2020). Temporomandibular Disorders: Priorities for Research and Care.

[20] Morgan M., Jenkins L., Ridsdale L. (2007). Patient pressure for referral for headache: A qualitative study of GPs’ referral behaviour. Br. J. Gen. Pract..

[21] Rozgony A.L. (2023). Facilitators and Barriers to Providing Orthodontic Care for Patients with Craniofacial Anomalies. Master’s Thesis.

[22] Broder H.S.R. (1991). Psychosocial problems and referrals among oral-facial team patients. J. Rehabil..

[23] Nelson P., Glenny A.M., Kirk S., Caress A.L. (2012). Parents’ experiences of caring for a child with a cleft lip and/or palate: A review of the literature. Child Care Health Dev..

[24] Association ACPC Standards for Approval of Cleft Palate and Craniofacial Teams—Revision. https://acpacares.org/wp-content/uploads/2023/01/Standards-for-Approval-2022.pdf.

[25] Razavi D., Delvaux N. (1997). Communication skills and psychological training in oncology. Eur. J. Cancer.

[26] Crerand C.E., Kapa H.M., Litteral J., Pearson G.D., Eastman K., Kirschner R.E. (2018). Identifying Psychosocial Risk Factors Among Families of Children with Craniofacial Conditions: Validation of the Psychosocial Assessment Tool-Craniofacial Version. Cleft Palate Craniofac J..

[27] Khondker A., Lee M.H., Kangasjarvi E., Simpson J.S. (2023). Educational exposures associated with preclinical medical student interest in pursuing surgical residency: Longitudinal mixed-methods study with narrative evaluation. Surg. Open Sci..

[28] Burford B., Whittle V., Vance G.H. (2014). The relationship between medical student learning opportunities and preparedness for practice: A questionnaire study. BMC Med. Educ..

[29] Kandiah D.A. (2017). Perception of educational value in clinical rotations by medical students. Adv. Med. Educ. Pract..

[30] Watters A.L., Stabulas-Savage J., Toppin J.D., Janal M.N., Robbins M.R. (2015). Incorporating Experiential Learning Techniques to Improve Self-Efficacy in Clinical Special Care Dentistry Education. J. Dent. Educ..

[31] Preston K., Chen L., Brennan T., Sheller B. (2023). Orthodontic treatment protocols in patients with alveolar clefting: A survey of ACPA-approved cleft teams in the United States. Angle Orthod..

[32] American Dental Association Predoctoral Dental Education Program Dashboard. https://www.ada.org/resources/research/health-policy-institute/dental-education/dental-school-trends-dashboard.

