# Supplementary material for: Identifying Gaps in Predoctoral Craniofacial Education
Source: Dent J (Basel). 2025 Jun 16;13(6):266. doi: 10.3390/dj13060266 (PMC12192217; doi:10.3390/dj13060266)
Supplement: Supplementary file 1 [file dentistry-13-00266-s001.zip › dentistry-3647761-supplementary Figure S1.pdf]

UNC SCHOOL OF DENTISTRY

Start of Block: Block 4

Please refer to the following definition of craniofacial differences to complete this survey:

Craniofacial differences = a range of congenital or acquired conditions that affect the structure and appearance of the head and face regions. These differences can include orofacial clefts, craniosynostosis, cleidocranial dysplasia, and various syndromes that impact craniofacial development.

End of Block: Block 4

UNC SCHOOL OF DENTISTRY

Start of Block: Section 1: Demographics

*

How old are you?

____

What is your gender identity?

- ○ Woman
- ○ Man
- ○ Transgender
- ○ Non-binary/non-conforming
- ○ Prefer not to respond

Which racial group(s) do you identify with? (select all that apply)

- ○ American Indian or Alaska Native
- ○ Asian
- ○ Black or African American
- ○ Native Hawaiian or Other Pacific Islander
- ○ White
- ○ Other (please specify) ____
- ○ Prefer not to respond

Which ethnic group do you identify with?

- ◯ Hispanic or Latino
- ◯ Not Hispanic or Latino
- ◯ Other (please specify) ____
- ◯ Prefer not to respond

What year of education are you currently in?

- ◯ D1
- ◯ D2
- ◯ D3
- ◯ D4

End of Block: Section 1: Demographics

Start of Block: Section 2: Educational and Clinical Encounters

Have you received any didactic teaching on the diagnostic characteristics of craniofacial differences at your dental school?

- ◯ Yes
- ◯ No

Have you received any didactic teaching on the psychosocial challenges that craniofacial patients face?

- Yes
- No

Carry Forward Selected Choices from "Q8"

X→

Have you received any teaching on how to treat and refer craniofacial patients at your dental school?

- Yes
- No
- Yes
- No

End of Block: Section 2: Educational and Clinical Encounters

Start of Block: Section 3: Perceived Knowledge & Confidence Levels

UNC SCHOOL OF DENTISTRY

With the training you have been provided, please rate your level of confidence with the following skills related to craniofacial differences

	0 10 20 30 40 50 60 70 80 90 100
Provide restorative treatment for craniofacial patients	
Provide surgical treatment (simple extraction) for craniofacial patients	
Have a craniofacial patient in your practice for regular care	
Differentiate between "when to treat" and "when to refer" craniofacial patients	
Refer to and communicate with other healthcare providers regarding patients with craniofacial differences (such as an speech language pathologist, physician, psychologist, etc)	
Order and interpret radiographs of patients with craniofacial differences	
Perform an occlusal analysis for craniofacial patients	
Educate patients and their families about the oral health implications of craniofacial differences	

Rate your level of understanding for the following craniofacial conditions

	0 10 20 30 40 50 60 70 80 90 100
Orofacial clefts (cleft lip and/or palate)	
Treacher Collins syndrome	
Craniosynostosis	
Cleidocranial dysplasia	
Pierre Robin sequence	
Hemifacial microsomia	
Apert syndrome	

How helpful would the following resources be in improving your confidence to provide care for craniofacial patients?

	0 10 20 30 40 50 60 70 80 90 100
More lectures	
Shadowing opportunities	
Clinical rotations in specialized centers	
Hands on clinical experience	
Online modules	
Guest speakers/experts in the field	
Additional textbooks/articles	

Please feel free to share your thoughts on anything you would like us to know about craniofacial education

____

End of Block: Section 3: Perceived Knowledge & Confidence Levels
