# Supplementary material for: Identifying Gaps in Predoctoral Craniofacial Education
Source: Dent J (Basel). 2025 Jun 16;13(6):266. doi: 10.3390/dj13060266 (PMC12192217; doi:10.3390/dj13060266)
Supplement: Supplementary file 1 [file dentistry-13-00266-s001.zip › dentistry-3647761-supplementary tables.pdf]

Table S1. Association between perceived confidence level and previous didactic teaching according to academic year.

**Survey Item**	**Academic year**	**Previous didactic teaching**								
		On diagnostic characteristics of craniofacial differences			On psychosocial challenges faced by craniofacial patients			On treatment and referral of craniofacial patients		
		Yes	No	P value	Yes	No	P value	Yes	No	P value
1. Provide restorative treatment for craniofacial patients	D1	10.9 ± 18.6	4.8 ± 6.2	.124	4.6 ± 3.6	8.4 ± 15.1	.509	28.2 ± 35.4	5.8 ± 8.2	.230
	D2	34.4 ± 23.6	27.9 ± 26.8	.446	35.3 ± 23.8	30.8 ± 24.8	.495	43.7 ± 24.7	29.3 ± 23.0	.046
	D3	52.3 ± 28.1	39.6 ± 28.3	.248	56.0 ± 27.1	44.7 ± 28.8	.152	62.0 ± 26.6	37.7 ± 24.7	.001*
	D4	50.4 ± 26.5	43.0 ± 4.2	.700	56.7 ± 25.6	41.0 ± 24.3	.039	50.8 ± 25.7	48.7 ± 27.3	.794
2. Provide surgical treatment (simple extraction) for craniofacial patients	D1	8.3 ± 15.8	4.0 ± 3.8	.186	4.6 ± 4.5	6.5 ± 12.4	.694	20.4 ± 33.6	4.7 ± 5.5	.356
	D2	25.8 ± 23.3	28.1 ± 24.9	.783	25.9 ± 21.6	26.6 ± 25.8	.917	32.9 ± 21.6	23.8 ± 23.8	.200
	D3	42.8 ± 28.2	35.0 ± 30.2	.477	45.7 ± 26.9	37.5 ± 29.7	.302	53.4 ± 27.6	28.9 ± 23.5	.001
	D4	50.2 ± 24.4	19.5 ± 14.8	.088	56.3 ± 25.9	38.9 ± 19.5	.015	49.1 ± 25.5	48.5 ± 24.2	.039
3. Have a craniofacial patient in your practice for regular care	D1	18.4 ± 23.1	7.1 ± 16.1	.043	8.7 ± 10.3	13.5 ± 21.8	.574	23.0 ± 31.4	11.8 ± 19.4	.251
	D2	38.4 ± 28.1	21.0 ± 21.3	.043	38.8 ± 28.2	30.9 ± 27.0	.297	53.4 ± 28.2	28.4 ± 24.5	.002
	D3	51.7 ± 29.5	37.1 ± 34.8	.216	57.0 ± 29.1	41.9 ± 30.5	.074	64.2 ± 26.7	33.6 ± 26.3	<.001*
	D4	50.7 ± 28.4	50.5 ± 14.8	.992	53.6 ± 30.0	46.8 ± 24.9	.409	49.2 ± 28.3	53.4 ± 27.7	.621
4. Differentiate between "when to treat" and "when to refer" craniofacial patients	D1	15.7 ± 18.9	7.0 ± 7.9	.036	13.4 ± 18.0	11.3 ± 14.8	.711	29.2 ± 27.7	9.6 ± 12.2	.189
	D2	38.6 ± 26.1	21.9 ± 26.6	.075	39.6 ± 26.8	30.6 ± 26.3	.213	50.9 ± 26.8	29.8 ± 24.6	.008
	D3	50.6 ± 25.2	36.5 ± 34.9	.175	52.8 ± 27.0	44.1 ± 26.8	.245	61.7 ± 23.3	34.2 ± 23.4	<.001*
	D4	59.5 ± 26.7	54.5 ± 48.8	.803	66.4 ± 24.0	49.7 ± 28.6	.035	57.5 ± 28.7	62.3 ± 24.5	.568
5. Refer to and communicate with other healthcare providers regarding patients with craniofacial differences	D1	29.8 ± 28.8	9.0 ± 17.2	.003	29.3 ± 36.8	18.1 ± 23.9	.290	42.8 ± 39.7	17.2 ± 23.2	.224
	D2	47.6 ± 24.8	20.3 ± 19.1	.002	48.6 ± 26.5	35.4 ± 23.9	.060	58.7 ± 28.2	36.6 ± 22.6	.004
	D3	54.7 ± 26.7	29.8 ± 31.4	.022	59.4 ± 25.8	42.2 ± 20.1	.029	62.1 ± 24.5	28.6 ± 28.1	.002
	D4	56.5 ± 27.3	28.0 ± 25.5	.155	63.9 ± 26.5	43.7 ± 25.1	.012	57.6 ± 29.9	51.1 ± 23.1	.442
6. Order and interpret radiographs of patients with craniofacial differences	D1	17.7 ± 18.2	6.0 ± 7.9	.004	8.9 ± 10.7	12.4 ± 15.8	.571	21.0 ± 23.7	11.0 ± 14.1	.163
	D2	32.4 ± 23.8	18.2 ± 14.5	.023	35.4 ± 23.2	23.1 ± 21.2	.047	52.1 ± 22.1	21.4 ± 17.0	<.001*
	D3	61.0 ± 29.1	41.8 ± 27.9	.090	62.4 ± 28.3	53.6 ± 30.6	.284	72.4 ± 23.2	42.5 ± 27.9	<.001*

	D4	51.3 ± 25.3	28.5 ± 26.2	.218	62.7 ± 20.8	33.8 ± 21.7	<.001*	53.2 ± 27.3	45.4 ± 21.8	.321
7. Perform an occlusal analysis of craniofacial patients	D1	15.8 ± 22.8	7.2 ± 14.4	.106	4.4 ± 4.1	12.7 ± 20.6	.301	26.4 ± 25.4	10.0 ± 18.4	.073
	D2	33.2 ± 25.3	15.4 ± 21.2	.044	36.9 ± 27.7	21.7 ± 19.7	.022	44.7 ± 26.9	24.4 ± 22.7	.007
	D3	50.6 ± 30.3	26.4 ± 30.7	.043	57.9 ± 29.7	35.9 ± 29.4	.010	63.0 ± 28.9	29.6 ± 23.9	<.001*
	D4	35.3 ± 26.8	28.0 ± 25.5	.707	45.8 ± 26.2	20.5 ± 19.4	<.001*	39.2 ± 27.6	27.7 ± 23.5	.157
8. Educate patients and their families about the oral health implications of craniofacial differences	D1	29.3 ± 28.9	8.6 ± 12.2	.002	36.7 ± 35.3	16.5 ± 21.7	.041	32.6 ± 35.0	17.8 ± 23.2	.200
	D2	41.5 ± 25.8	16.3 ± 20.3	.006	42.8 ± 26.4	29.9 ± 25.6	.073	56.3 ± 23.9	29.7 ± 24.0	<.001*
	D3	51.6 ± 25.4	25.1 ± 24.3	.009	57.8 ± 25.8	37.2 ± 24.0	.004	60.6 ± 24.2	33.3 ± 22.1	<.001*
	D4	44.9 ± 27.1	48.5 ± 54.4	.860	55.6 ± 24.7	30.8 ± 25.4	.002	45.4 ± 27.1	44.5 ± 29.6	.913

*denotes statistically significant differences after adjusting for Holm-Bonferroni correction.

Table S2. Correlation matrix between levels of confidence and understanding.

	1	1.1	1.2	1.3	1.4	1.5	1.6	1.7	1.8	2	2.1	2.2	2.3	2.4	2.5	2.6	2.7
1.	1																
1.1	0.8804804	1															
1.2	0.8317508	0.8319149	1														
1.3	0.8588128	0.8036409	0.694249	1													
1.4	0.8538233	0.6997767	0.6759201	0.7175761	1												
1.5	0.8486335	0.6551687	0.6471414	0.675149	0.6850982	1											
1.6	0.8543233	0.7144736	0.6878247	0.6419157	0.7068842	0.6641494	1										
1.7	0.8191195	0.6577125	0.5906849	0.6662427	0.6122242	0.6359839	0.694481	1									
1.8	0.8044871	0.587499	0.5029539	0.5876805	0.6580825	0.7591433	0.6580366	0.6803082	1								
2.	0.7745517	0.6650383	0.6129431	0.6371597	0.702117	0.6790862	0.6632978	0.6087627	0.6590078	1							
2.1	0.6196145	0.4585613	0.4328938	0.5298531	0.5451891	0.5904667	0.5282756	0.5105901	0.5821348	0.7780091	1						
2.2	0.7151183	0.5942336	0.5605195	0.5593954	0.6462239	0.6712481	0.6003495	0.5428565	0.6517837	0.8804125	0.7262429	1					
2.3	0.744368	0.6582601	0.5681314	0.6111376	0.644507	0.6256096	0.659791	0.6092077	0.6482285	0.8832877	0.6096891	0.7417158	1				
2.4	0.6756386	0.6057365	0.5062759	0.5343996	0.5760709	0.588233	0.6153162	0.549425	0.5850989	0.8897373	0.6525426	0.7916138	0.7970759	1			
2.5	0.6145358	0.5400636	0.5740253	0.5061183	0.6090611	0.5436896	0.4796898	0.4363452	0.4600748	0.8175241	0.4991376	0.6533272	0.6327303	0.6377528	1		
2.6	0.6201508	0.5599411	0.4677117	0.5310971	0.5641541	0.4827254	0.5513393	0.5189174	0.5093134	0.8701604	0.6017303	0.6683667	0.7866654	0.7663722	0.6476286	1	
2.7	0.6080147	0.5364431	0.5452268	0.5120395	0.5914108	0.5237989	0.4957048	0.43768	0.4617198	0.815572	0.4778846	0.6204034	0.6846254	0.6025557	0.8282859	0.6923993	1
	1. Overall confidence level	1.1 Provide restorative treatment for craniofacial patients	1.2 Provide surgical treatment (simple extraction) for craniofacial patients	1.3 Have a craniofacial patient in your practice for regular care	1.4 Differentiate between "when to treat" and "when to refer" craniofacial patients	1.5 Refer to and communicate with other healthcare providers regarding patients with craniofacial differences	1.6 Order and interpret radiographs of patients with craniofacial differences	1.7 Perform an occlusal analysis of craniofacial patients	1.8 Educate patients and their families about the oral health implications	2. Overall understanding	2.1 Understanding of orofacial clefts (cleft lip and/or palate)	2.2 Understanding of Treacher Collins syndrome	2.3 Understanding of craniosynostosis	2.4 Understanding of cleidocranial dysplasia	2.5 Understanding of Pierrer Robin sequence	2.6 Understanding of hemifacial microsomia	2.7 Understanding of Apert syndrome

Table S3. Logistic regression analysis to predict sufficient overall confidence level (above 50% threshold) according to academic year.

**Predictors**			**Beta**	***SE***	**Wald**	***p* value**	**OR**	**95% CI LL - UL**
SECOND YEAR STUDENTS								
Overall understanding		Self-perceived level of understanding	0.09	0.03	8.65	.003*	1.10	1.03, 1.17
Previous didactic teaching	Survey item	Diagnostic characteristics of craniofacial differences	-0.29	1.42	0.04	.837	1.34	0.08, 21.45
		Psychosocial challenges faced by craniofacial patients	-0.03	1.06	0.00	.977	1.03	0.13, 8.25
		Treatment and referral of craniofacial patients	3.25	1.21	7.25	.007*	0.04	0.00, 0.41
THIRD YEAR STUDENTS								
Overall understanding		Self-perceived level of understanding	0.09	0.03	9.74	.002*	1.09	1.03, 1.16
Previous didactic teaching	Survey item	Diagnostic characteristics of craniofacial differences	1.33	1.71	0.60	.437	0.26	0.01, 7.56
		Psychosocial challenges faced by craniofacial patients	-1.47	1.04	2.03	.154	4.37	0.57, 33.19
		Treatment and referral of craniofacial patients	1.91	0.87	4.80	.029*	0.15	0.03, 0.82
FOURTH YEAR STUDENTS								
Overall understanding		Self-perceived level of understanding	0.08	0.03	8.61	.003*	1.08	1.03, 1.14
Previous didactic teaching	Survey item	Diagnostic characteristics of craniofacial differences	-1.59	1.70	0.87	.351	4.89	0.17, 135.43
		Psychosocial challenges faced by craniofacial patients	0.95	0.83	1.31	.253	0.39	0.08, 1.97
		Treatment and referral of craniofacial patients	0.80	0.81	0.97	.325	0.45	0.09, 2.22

Note. *p <.05. The analysis did not include first-year students for the small number of students with prior training.
CI: confidence interval;
LL: lower limit;
OR: odds ratio;
UL: upper limit.
